# Supplementary material for: Fine root and soil carbon stocks are positively related in grasslands but not in forests
Source: Commun Earth Environ. 2025 Jul 1;6(1):497. doi: 10.1038/s43247-025-02486-9 (PMC12213611; doi:10.1038/s43247-025-02486-9)
Supplement: Supplementary file 2 — Description of Additional Supplementary Files [file 43247_2025_2486_MOESM2_ESM.docx]

**Description of Additional Supplementary Files**

File name- Supplementary Data 1

File description - This R code processes and analyzes NEON megapit data to study carbon stocks in soil organic matter (SOC) and below-ground biomass (BGB) across various environmental sites. It aligns root and soil data by depth, calculates cumulative carbon fractions, and integrates environmental covariates like temperature and precipitation. Using mixed-effects models, it evaluates correlations between SOC and root carbon stocks for whole profiles and horizon-specific layers across forest and grassland ecosystems.
